# Scaling of decoherence for a system of uncoupled spin qubits

**DOI:** 10.1038/srep17013

**Published:** 2015-11-23

**Authors:** Jun Jing, Xuedong Hu

**Affiliations:** 1 Department of Physics, University at Buffalo, SUNY, Buffalo, NY 14260, USA; 2 Institute of Atomic and Molecular Physics, Jilin University, Changchun 130012, Jilin, China

## Abstract

Significant experimental progresses in recent years have generated continued interest in quantum computation. A practical quantum computer would employ thousands if not millions of coherent qubits, and maintaining coherence in such a large system would be imperative for its utility. As an attempt at understanding the quantum coherence of multiple qubits, here we study decoherence of a multi-spin-qubit state under the influence of hyperfine interaction, and clearly demonstrate that the state structure is crucial to the scaling behavior of *n*-spin decoherence. Specifically, we find that coherence times of a multi-spin state at most scale with the number of qubits *n* as 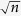, while some states with higher symmetries have scale-free coherence with respect to *n*. Statistically, convergence to these scaling behavior is generally determined by the size of the Hilbert space *m*, which is usually much larger than *n* (up to an exponential function of *n*), so that convergence rate is very fast as we increase the number of qubits. Our results can be extended to other decoherence mechanisms, including in the presence of dynamical decoupling, which allow meaningful discussions on the scalability of spin-based quantum coherent technology.

Large-scale quantum information processing (QIP) requires the generation, manipulation, and measurement of fully coherent superposed quantum states involving many qubits^1^. A central issue for QIP is how well such a many-qubit system can maintain its quantum coherence. From the perspective of fundamental physics, an equally intriguing question that has been repeatedly asked is how a large number of microscopic quantum mechanical systems together behave classically as a macroscopic object^2^. To answer these questions, it is crucial to identify the key elements determining the scaling behavior of the decoherence of a multi-qubit system.

A confined single electron spin in a semiconductor quantum dot (QD) or a shallow donor is highly quantum coherent, and is a promising candidate as a qubit^3^,^4^,^5^,^6^,^7^,^8^,^9^,^10^,^11^,^12^. It is now well understood that the main single-spin decoherence channel is through hyperfine coupling to the environmental nuclear spins^10^,^12^,^13^, and the effects of hyperfine interaction have been investigated for coupled two- and three-spin systems^14^,^15^,^16^,^17^,^18^,^19^,^20^,^21^,^22^. A many-spin-qubit system thus offers a convenient test ground for studying decoherence scaling since different factors in the overall decoherence can be easily distinguished.

The study of whether quantum coherent features of a many-qubit system can survive over long evolution times started with the discovery and exploration of the decoherence-free subspace (DFS)^23^,^24^,^25^,^26^,^27^, where the many qubits in a system share a common reservoir. The states in a DFS do not experience decoherence from the collective noise from the reservoir, while states outside the subspace do. The concept of DFS clearly illustrates an important difference between decoherence of a single qubit and that for many qubits: the decoherence of single-qubit is characterized by relaxation time *T*_1_ and dephasing time *T*_2_, *irrespective of the qubit state*; while with the many more density matrix elements involved, the decoherence of an *n*-qubit state is generally state-structure-dependent. This dependence is the main focus of the present work.

In this study we focus on the hyperfine-induced decoherence of *n* (≫1) uncoupled QD-confined electron spin qubits. Our goals are to clarify how decoherence of many-qubit states depends on the number of qubits and the state structure. In our study, a uniform magnetic field is applied to make the Zeeman splitting Ω much larger than the nuclear-spin-induced inhomogeneous broadening (see Fig. 1), so that spin relaxation is negligible. The dominant single-spin decoherence channel is pure dephasing due to the nuclear spins. We explore how this mechanism affects a many-spin-qubit state by systematically examining a large number of superposed states in various forms. Specifically, if the fidelity of an *n*-qubit state decays as exp[−γ(*t*)], we clarify how *γ*(*t*) depends on the qubit number *n* or the number of basis states *m* (which could be exponentially large as compared to *n*). Our results from this broad-ranged exploration indicate decoherence scaling behavior ranging from scale-free up to sublinear to *n*, making the scale-up of a spin-based quantum computer a tractable endeavor.

## Electron-nuclear spin hyperfine interaction

We consider *n* uncoupled electron spins in a finite uniform magnetic field, each confined (in a quantum dot, nominally) and interacting with its own uncorrelated nuclear-spin bath through hyperfine interaction:


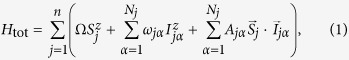


where 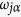 is the nuclear Zeeman splitting of the *α*-th nuclear spin in the *j*-th QD (from here on *j* will always be used to label the QDs and the corresponding electron spin qubits), and *A*_*ja*_ is the hyperfine coupling strength. The number of nuclear spins coupled to the *j*-th electron spin, *N*_*j*_, is in the order of 10^5^ to 10^6^ in GaAs QDs, and 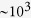 in natural Si QDs.

The total Hamiltonian (1) is a sum of *n* fully independent single-spin decoherence Hamiltonians. The evolution operator for these *n* qubit can thus be factored into a product of operators for individual qubits. We present a brief recap of single-spin decoherence^13^,^28^ properties in Method, and focus here on the multi-spin-qubit decoherence problem. Recall that inhomogeneous broadening corresponds to stochastic phase diffusion of an electron spin due to longitudinal Overhauser field, and is characterized by the time scale 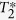. On the other hand, the narrowed-state free induction decay is caused by fluctuations in the transverse Overhauser field, and is characterized by the time scale *T*_2_. These two time scales are statistically independent because of independence between longitudinal and transverse Overhauser fields, as presented in Method. These two pure dephasing channels follow the same scaling law, i.e., 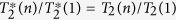, where *n* is the number of spin qubits in the system. Thus we can focus on the scaling analysis of either of them. In the following we employ 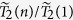 to represent the result, which is applicable to both dephasing channels.

## Results

### Multi-spin decoherence

**Figure 1:**
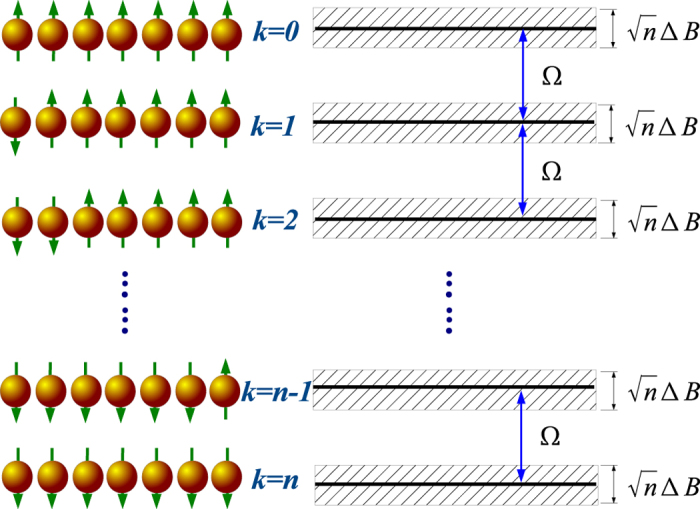
The energy spectrum for *n* electron spins separately confined in *n* uncoupled QDs in a finite uniform magnetic field. The spectrum splits into *n* + 1 Zeeman sub-levels. *k* refers to the number of spins that point down. Each electron spin is coupled to local nuclear spins through hyperfine interaction, which produces a local field in the order of ∆*B*, so that the energy level for each Zeeman manifold is broadened to a band with width 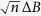.

For an *n*-spin system in a finite uniform magnetic field, the full Hilbert space is divided into *n*+1 Zeeman subspaces, labeled by 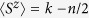, 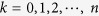. Each subspace consists of 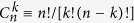 degenerate states (in the absence of nuclear field), which has *k* spins in the 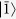 (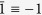) state and 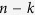 spins in the 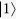 state. The local random Overhauser fields break this degeneracy and lead to a broadening of the manifold 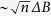 (see Fig. 1). In all the following calculations, we use spin product states 
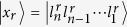
 as the bases. Here 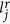 refers to the electron spin orientation along the *z*-direction in the *j*-th QD for state 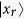, and takes the value of 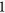 or 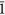 for notational simplicity.

For a superposed state 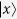 containing more than one product state, decoherence emerges due to the non-stationary random phase differences among the *m* product states 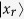
's: 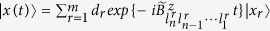 with 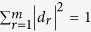. The number of product states in 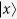, 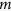, is also the Hilbert space size of concern because spin relaxation is generally negligible in a finite field and is not considered in this study. We treat the Overhauser field (both longitudinal and transverse components) semiclassically, accurate to the second order in its magnitude. The notation 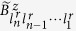 represents a sum of Overhauser fields from every QD, and is defined in Method. As a measure of decoherence of 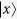 caused by the hyperfine interaction, we use fidelity 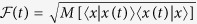, which can be simplified in the presence of dephasing as


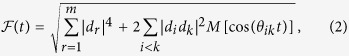


where 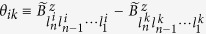 is the Overhauser field difference experienced by the two *n*-spin product states (see Method). Specifically, 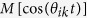 is solely determined by the number of spins that are opposite in orientation between bases 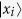 and 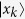. Therefore, the fidelity depends on the structure of the interested state, i.e., the constituents and their weight in the superposed state, and single-qubit decoherence is only one of several important ingredients in the multi-qubit decoherence problem.

### Classification of multi-spin decoherence

With our understanding of single-spin decoherence, and with fidelity of the collective decoherence for a multi-spin state 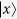 defined, we are now in position to clarify multi-spin decoherence in various subspaces of the *n*-spin system.

### Case A: single product state

The simplest multi-spin state is a single product state. The random Overhauser fields experienced by the spin qubits create a random but global phase (relative to when the nuclear reservoir is absent). This global phase does not lead to any decoherence, as there is no coherence (phase) information stored in any product state.

### Case B: two product states, with *m* = 2 and *k* 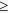


The simplest multi-spin state that can undergo dephasing consists of two product states. Here we choose a particular class of 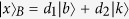, with one state being fully polarized 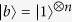, while the other being from the *k*-th subspace with *k* spins in 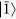. The fidelity of this state is 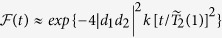, so that


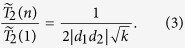


In this case, dephasing time is inversely proportional to the square root of the number of spins prepared as 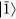 in 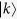. A special example here is the GHZ state, 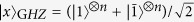. The decoherence rate is 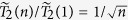, where the square root of the number of spin qubits is from the quadratic time dependence in the exponent of 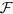. The worst case scenario for an 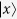 containing two product states is when they have completely opposite spins.

### Case C: *n* ≥ *m* ≥ 2, *k* = 1 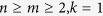


We now consider an 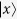 that is a superposition of *m* product states from the manifold with one spin in 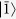. Explicitly, 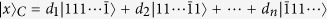, where 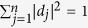. This state is slightly more general than the well-known *W* state, with a random weight and phase for each basis state. The fidelity of 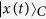 is 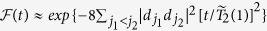, which implies (by the Cauchy
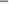
Schwarz inequality)


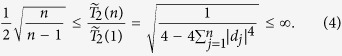


Here the upper bound (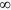 means no decoherence) is approached when a particular product state dominates over all others in weight: 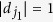 while 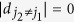, so that we go back to **Case A**. The lower bound for decoherence time is scale-free with respect to *n*, when the whole system acts like a giant spin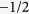 system in which the spin polarization is spread out over *n* physical spins. The lower bound corresponds to the equally-populated superposed states with 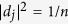, i.e., an almost standard *W* state (which would have all *d*_*j*_ having the same phase, too). For a large number of qubits, 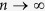, 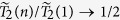, where the scaling of decoherence is insensitive to either the population distribution on each basis state or the total number of physical spins.

### Case D: m = 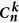


We now extend 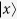 to a more generalized *W*-state that is distributed over all the product bases in the *k*-th Zeeman manifold, with 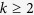. For a clear physical picture let us first consider a special example where all the product states have the same weight: 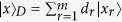 with 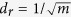. The overall decoherence is determined by the phase differences between every pair of states from the 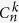 basis states as well as the population distribution. Since 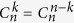, we limit our discussion below to 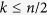 without loss of generality. The phase difference 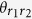 between a particular pair of 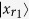 and 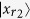 can involve Overhauser fields in 2*j* QDs, where 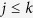. In the extreme case of 2*j* = *n*, they have completely opposite spins. After a straightforward derivation via combinatorial mathematics, the fidelity for this state is found to be 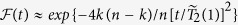. Thus


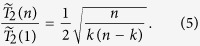


In particular, (a) when 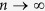 while *k* is kept as a constant, 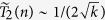, which is scale-free with respect to the number of spins *n* as well as the number of product states *m* in 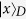 (it is a similar feature as in **Case C** with 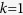); (b) overall decoherence is completely suppressed when 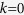 or 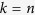, i.e. 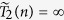. These two Zeeman manifolds contain one state each, so that **Case D** is reduced to **Case A**; (c) the strongest decoherence occurs when 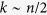, where 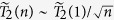.

**Figure 2:**
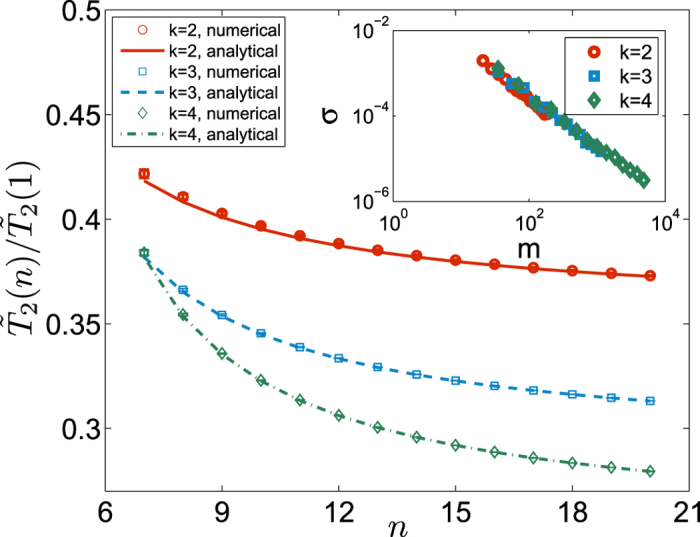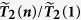 vs. *n* for randomly generated 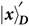 states (with random populations over bases) in the 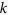-th Zeeman manifold in Case D. The lines are generated from the analytical expression of Eq. (5) based on the 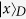 state. Inset: standard deviation *σ* of 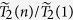 obtained from 100 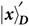 states, as a function of the Hilbert space size *m*.

The generalized *W* state 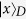 here is a reliable and tight lower bound for the decoherence scaling rate of a more general state 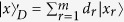 in the *k*-th manifold where *d*_*r*_ is an arbitrary number. In Fig. 2, the lines represent the analytical result given by Eq. (5), and the data points are obtained from 100 randomly generated 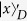 states. The inset of the figure shows that the standard deviations in 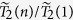 for the random 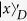 states scale as a power-law function of *m*. More specifically, 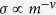, when 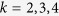, 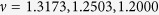, respectively. This *m*-dependence originates from the randomness we have introduced in the populations of the *m* states involved in each 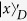. With 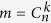, the convergence of the calculated 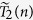 is extremely fast as we increase *n*, as indicated in Fig. 2. In short, Fig. 2 clearly indicates that the equal-weight 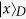 state is a very good representative of the large class of states from both **Cases C** and **D**. Furthermore, while decoherence rate of 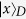 generally scales as 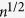, the convergence rate scales as 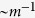.

### Case E: *m* = 2^*n*^

We now consider 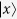 in the full Hilbert space of the *n* qubits. For the overall decoherence, 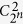 pairs of phase differences have to be taken into account. The simplest such state is the fully and equally superposed state 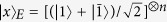, which is the initial state employed by Shor’s algorithm of factorization^29^ and one-way computing^30^. Its fidelity is simply the product of single-qubit fidelity 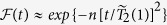. Thus,


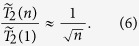


**Figure 3:**
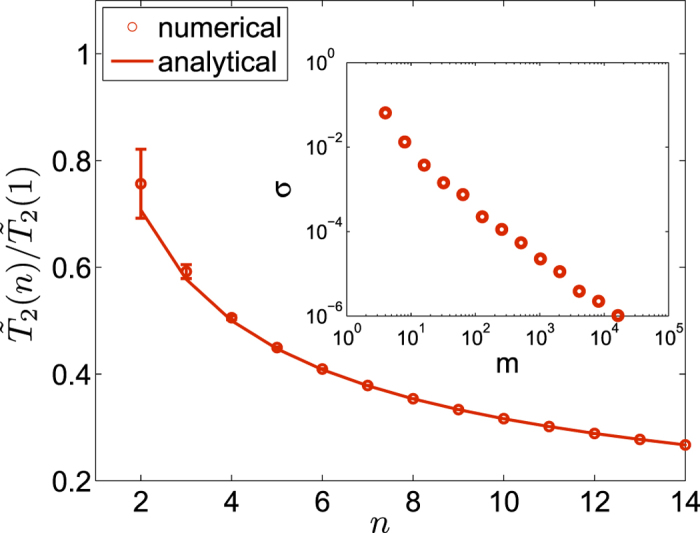
Average 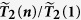 vs. *n* from randomly generated states over the whole Hilbert space of the *n*-spin system. The solid line is generated by Eq. (6), using the equal-superposition state 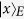. Inset: standard deviation of 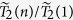 vs. Hilbert space size *m* = 2^*n*^. For each *n*, The results are generated from 100 randomly selected states.

As in **Case D**, we can generalize 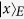 to 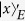 by randomizing the weight 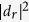
's, 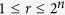. In Fig. 3 we plot our numerical results as compared with the analytical expression from Eq. (6). The size of error bars in Fig. 3 for random states rapidly vanishes with increasing *n*. Similar to **Case D**, the inset shows that the standard deviation of 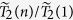 scales with the Hilbert space size *m* in the form 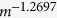. Since here *m* increases exponentially with *n*, the rapid suppression of error bar size as we increase *n* is not surprising. Consequently, the decoherence time for an arbitrary state 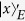 adheres to the sublinear power-law 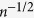 as soon as 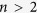.

## Discussion

We have explored the scaling behavior of decoherence of *n* uncoupled electron spin qubits by investigating the fidelity of 5 classes of representative superposed states 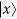. Our results are summarized in Table 1, where *k* is the number of spins in 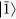 in a product state that makes up of 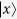. Typically, the pure dephasing rates are not related to the sub-Hilbert-space size *m*. Instead, they are usually sublinear power-law functions of the qubit number *n*, with the exponent determined by the single-spin decoherence mechanism. Furthermore, if 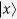 is constrained in a single subspace with a fixed *k*, 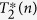 and 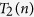 become scale-free with respect to *n* and *m*, in the spirit of DFS, though the noise sources here are not common to all qubits.

Fidelity is one specific way to represent the environmental decoherence effects on a multi-qubit state, with equally weighted contributions from all the off-diagonal density matrix elements. We choose it partially because there is no consensus measure for multi-qubit entanglement. Still, fidelity does provide hints on the robustness of certain entangled states against pure dephasing considered in this study. It should be noted that the results for the often-studied multipartite states, GHZ states and *W* states (presented in **Cases B** and **C**, respectively) coincide with their entanglement behaviors. The entanglement of *W* states (fidelity undergoes scale-free decay with respect to *n*) outperforms that of GHZ states (fidelity decay rate is proportional to 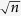) in terms of their robustness^31^. The independence on *n* by the *W* states is generic, insensitive to the behavior of single-qubit decoherence.

The scalings revealed in our case studies can be qualitatively understood by counting the number of different spin orientations in any pair of product states. Among *m* product states making up an arbitrary state 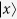, a large fraction of pairs have 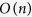 electron spins oriented in the opposite direction. If we average over all possible states assuming 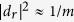, the fidelity given by Eq. (2) could be estimated as 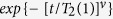. The decoherence rates are insensitive to *m* because of normalization and our equal-population assumption. More specifically, in the *k*-th manifold, the scaling law is 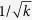 because any pair of states here is different at most in 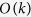 spins. This scale-free behavior (with respect to *n* and *m*) is quite generic^26^,^27^, and *not* dependent on single-qubit decoherence.

Our study here could be straightforwardly extended to other single-qubit decoherence mechanisms. In general, if the single-spin decoherence function is given by 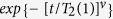, the index of every power-law (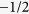) in Table 1 should be changed to 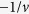. For decoherence due to Gaussian noise under dynamical decoupling^32^, the decay functions have 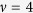 for spin echo and 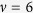 for two-pulse Carr-Purcell-Meiboom-Gill sequence, so that the decoherence scaling factors for the *n*-spin system become 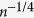 and 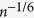, respectively. For spin relaxation induced by electron-phonon interaction that produces a linear exponential decay characterized by *T*_1_, the sub-Hilbert space spanned by a multi-qubit state is usually not fixed. So that a comprehensive understanding of the decay scaling power-laws requires further studies. Nevertheless, certain coherence terms in the *n*-spin system will still follow 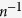 scaling, same as what our dephasing study indicates.

Generally, decoherence of any class of multi-qubit states is independent of the Hilbert space size *m*. Whether it is scale-free or scales as a polynomial of *n* depends on the state-structure, while the specific power-law depends on the single-qubit decoherence mechanism. On the other hand, the variability of decoherence for arbitrary states decreases polynomially with increasing *m* because we only consider dephasing.

In conclusion, we find that the structure of a multi-qubit state is a critical ingredient in determining its collective decoherence. While different from DFS^33^, the scale-free states help identify Hilbert subspaces that are more favorable in coherence preservation for spin-based qubits under the influence of local nuclear spin reservoirs.

## Method

### Single-Spin Decoherence

For a single electron spin coupled to the surrounding nuclear spins in a finite magnetic field, the nuclear reservoir causes pure dephasing via the effective Hamiltonian^13^,^28^


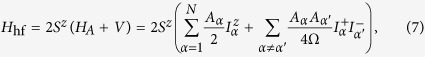


where *N* is the number of nuclear spins, Ω is the electron Zeeman splitting, and *A*_*α*_ is the hyperfine coupling strength. The sums over *α* and 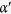 here are over all the nuclear spins in the single quantum dot (QD). The dephasing dynamics has two contributions: *H*_*A*_ is the longitudinal Overhauser field, while *V* is the second-order contribution from the transverse Overhauser field. In a finite field, normally the former dominates, generating a random effective magnetic field of 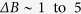 mT^9^ on a quantum-dot-confined electron spin in GaAs. This random field leads to a stochastic phase and accounts for the inhomogeneous broadening effect characterized by a free induction decay at the time scale of 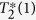, where 1 indicates that only one electron spin is considered. For this single spin, the inhomogeneous broadening decoherence function is:


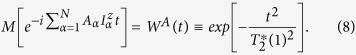


Here 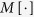 is an ensemble average over the longitudinal Overhauser field in the QD, and 
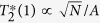
 with 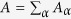. In a single gated QD in GaAs, 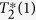 is in the order of 10 ns.

If the effect of *H*_*A*_ is suppressed, such as through nuclear spin pumping and polarization^10^, *V*, which is second order in the transverse Overhauser field, leads to the so-called narrowed-state free induction decay, by which the off-diagonal elements of the spin density matrix decay at the time scale of 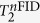. In the manuscript and here we will simplify the notation for 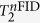 to 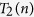, where *n* indicates the number of spin qubits in consideration. For a single spin, *n* = 1, and the narrowed-state decoherence function is given by:


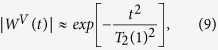


where 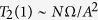
13, and is in the order of *μ*s in a gated GaAs QD.

### Notations on the multi-quantum-dot Overhauser fields

A convenient way to understand the effect of hyperfine interaction on the *n*-uncoupled-qubit system [see Eq. (1)] is to introduce the semiclassical Overhauser field: 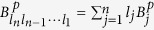, where 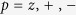 refers to the longitudinal and transverse directions, *l*_*j*_ takes the value of 1 or 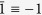, and 
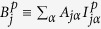
 is the Overhauser field in the *j* th QD. In a finite field and up to second order, the hyperfine Hamiltonian could be diagonalized on the product state basis into


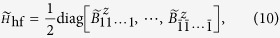


where


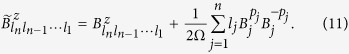


Here 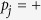 (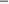) if 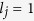 (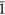). The two terms in Eq. (11) are responsible for the inhomogeneous broadening and narrowed-state FID, respectively. Accurate to the first order in 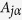, 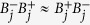, since 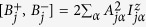 is second order in the hyperfine coupling strength and is small. For simplicity we take 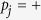 in the following derivation. Generally, the second-order term for the *j* th dot in Eq. (11) 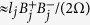. For example, a completely polarized state 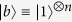 experiences a longitudinal Overhauser field 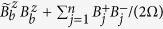. Thus our work is accurate to the second order of the hyperfine coupling. In the main text, the Overhauser fields are treated semiclassically, with the field operators replaced by c-numbers.

With the hyperfine Hamiltonian takes on a diagonal form, it only leads to dephasing between different product states due to 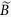, similar to the single-spin case we discussed above. The dephasing of a product state 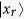 relative to 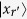 is due to the difference in the random Overhauser field 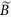 for these states.

### Statistical independence of inhomogeneous broadening and narrowed-state free induction decay

To analyze the relationship between inhomogeneous broadening from the longitudinal Overhauser field and narrowed-state free induction decay due to the transverse Overhauser field in an *n*-uncoupled-qubit system, we consider an arbitrary pure state in a subspace spanned by *m* spin product states 
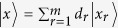
, where 
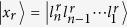
. Here 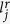 refers to the electron spin orientation along the *z*-direction in the *j* th QD for state 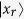, and takes the value of 1 or 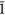 for notational simplicity. The whole Hilbert space of the *n*-qubit system could be divided to 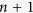 manifolds according to the number of 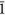 for the product bases, as indicated in Fig 1. The choice of 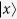 here is sufficiently general to cover all the cases discussed in the manuscript. Helped by the Overhauser fields defined above, and under the diagonalized hyperfine interaction Hamiltonian in Eq. (10), an initial state 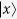 evolves into


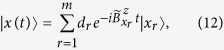


where 
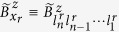
 is the Overhauser field experienced by the product state 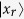. Decoherence of 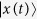 emerges due to the non-stationary random phase differences from these Overhauser fields. The fidelity between 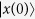 and 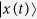 can be expressed as


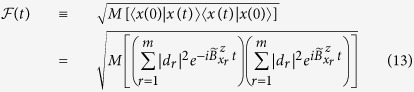



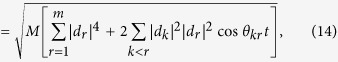


where the phase differences 
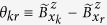
.

According to Eq. (11), each 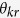 could be decomposed into two terms, 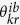 and 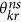, that are responsible for the inhomogeneous broadening and narrow-state free induction decay, respectively:


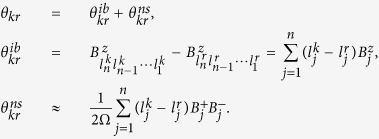


The ensemble average 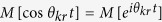 could be estimated using the decoherence times of a single qubit system 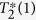 (inhomogeneous broadening time scale) and 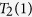 (the narrowed-state FID time scale)^13^,


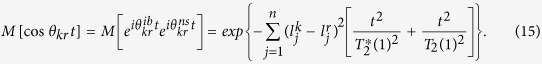


This result is obtained using the canonical approach to treat quantum noise^34^, and is valid at least in the short time limit. Physically it is based on the assumption that longitudinal and transverse Overhauser fields are independent from each other, so that the averages above can be factored. The two decoherence mechanisms are thus mutually independent. Using the short notations 
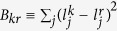
, 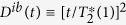, and 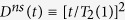, Eq. (14) can be rewritten as


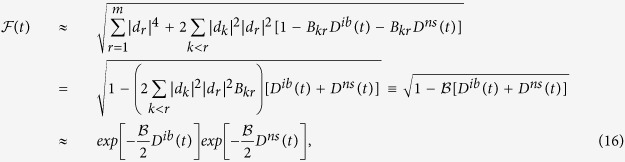


where 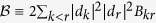. In short, Eqs (15) and (16) show that inhomogeneous broadening and narrowed-state FID are independent decoherence channels, and have the same scaling behavior. The overall decoherence function is just a simple product of the decay functions for inhomogeneous broadening FID and narrowed-state FID. We can thus focus on calculating 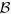 in our discussion of decoherence scaling for *n* spin qubits.

For a simple example, take 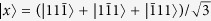. The inhomogeneous broadening part in Eq. (16) then takes on the form 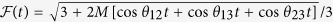, where 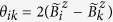. After a semiclassical evaluation of the Overhauser field noise^34^, and using the expressions of 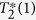 in Eq. (8) and 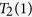 in Eq. (9), we find 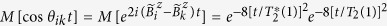, so that 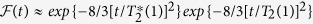 in the short-time limit. Therefore, in this example, 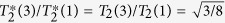.

## Additional Information

**How to cite this article**: Jing, J. and Hu, X. Scaling of decoherence for a system of uncoupled spin qubits. *Sci. Rep.* **5**, 17013; doi: 10.1038/srep17013 (2015).

## Figures and Tables

**Table 1:** A summary of decoherence times of *n* uncoupled electron spin qbits under the influence of hyperfine coupling with local nuclear baths.

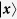	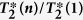 or 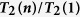
Stable: *A*	no decoherence
Two product states: *B*	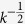
*k*-th subspace: *C* and *D*	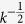
Crossing subspaces: *E*	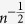

## References

[b1] Nielsen M. A. & Chuang I. L. Quantum computation and quantum information (Cambridge University Press, Cambridge, 2000).

[b2] Zurek W. H. Decoherence and the Transition from Quantum to Classical, Phys. Today, 44(10), 36 (1991).

[b3] Loss D. & DiVincenzo D. P. Quantum computation with quantum dots, Phys. Rev. A 57, 120 (1998).

[b4] Hanson R., Kouwenhoven L. P., Petta J. R., Tarucha S. & Vandersypen L. M. K. Spins in few-electron quantum dots, Rev. Mod. Phys. 79, 1217 (2007).

[b5] Rashba E. I. & Efros A. I. L. Orbital Mechanisms of Electron-Spin Manipulation by an Electric Field, Phys. Rev. Lett. 91, 126405 (2003). 14525382 10.1103/PhysRevLett.91.126405

[b6] Golovach V. N., Khaetskii A. & Loss D. Phonon-Induced Decay of the Electron Spin in Quantum Dots, Phys. Rev. Lett. 93, 016601 (2004).

[b7] Amasha S. *et al.* Electrical Control of Spin Relaxation in a Quantum Dot, Phys. Rev. Lett. 100, 046803 (2008). 18352316 10.1103/PhysRevLett.100.046803

[b8] Morello A. *et al.* Single-shot readout of an electron spin in silicon, Nature 467, 687 (2010). 20877281 10.1038/nature09392

[b9] Petta J. R. *et al.* Coherent Manipulation of Coupled Electron Spins in Semiconductor Quantum Dots, Science 309, 2180 (2005). 16141370 10.1126/science.1116955

[b10] Bluhm H. *et al.* Dephasing time of GaAs electron-spin qubits coupled to a nuclear bath exceeding 200*μ*s, Nat. Phys. 7, 109 (2011).

[b11] Pla J. J. *et al.* A single-atom electron spin qubit in silicon, Nature 489, 541 (2012). 22992519 10.1038/nature11449

[b12] Muhonen J. T. *et al.* Storing quantum information for 30 seconds in a nanoelectronic device, Nat. Nano. 9, 986 (2014). 10.1038/nnano.2014.211 25305745

[b13] Cywiński Ł., Witzel W. M. & Das Sarma S. Pure quantum dephasing of a solid-state electron spin qubit in a large nuclear spin bath coupled by long-range hyperfine-mediated interactions, Phys. Rev. B 79, 245314 (2009).

[b14] Coish W. A. & Loss D. Singlet-triplet decoherence due to nuclear spins in a double quantum dot, Phys. Rev. B 72, 125337 (2005).

[b15] Yang W. & Liu R. B. Quantum many-body theory of qubit decoherence in a finite-size spin bath, Phys. Rev. B 78, 085315 (2008).

[b16] Hung J. T., Cywiński Ł., Hu X. & Das Sarma S. Hyperfine interaction induced dephasing of coupled spin qubits in semiconductor double quantum dots, Phys. Rev. B 88, 085314 (2013).

[b17] Dial O. E. *et al.* Charge Noise Spectroscopy Using Coherent Exchange Oscillations in a Singlet-Triplet Qubit, Phys. Rev. Lett. 110, 146804 (2013). 25167023 10.1103/PhysRevLett.110.146804

[b18] Ladd T. D. Hyperfine-induced decay in triple quantum dots, Phys. Rev. B 86, 125408 (2012).

[b19] Medford J. *et al.* Quantum-Dot-Based Resonant Exchange Qubit, Phys. Rev. Lett. 111, 050501 (2013). 23952375 10.1103/PhysRevLett.111.050501

[b20] Mehl S. & DiVincenzo D. P. Noise-protected gate for six-electron double-dot qubit, Phys. Rev. B 88, 161408(R) (2013).

[b21] Hung J. T., Fei J., Friesen M. & Hu X. Decoherence of an exchange qubit by hyperfine interaction, Phys. Rev. B 90, 045308 (2014).

[b22] Kim D. *et al.* Quantum control and process tomography of a semiconductor quantum dot hybrid qubit, Nature 511, 70 (2014). 24990747 10.1038/nature13407

[b23] Palma G. M., Suominen K.-A. & Ekert A. K. Quantum Computers and Dissipation, Proc. Roy. Soc. London Ser. A, 452, 567 (1996).

[b24] Duan L.-M. & Guo G.-C. Reducing decoherence in quantum-computer memory with all quantum bits coupling to the same environment, Phys. Rev. A 57, 737 (1998).

[b25] Lidar D. A., Chuang I. L. & Whaley K. B. Decoherence-Free Subspaces for Quantum Computation, Phys. Rev. Lett. 81, 2594 (1998).

[b26] Buchleitner A., Viviescas C. & Tiersch M. (Eds.), Entanglement and Decoherence: Foundations and Modern trends (Springer-Verlag Berlin Heidelberg 2009).

[b27] Breuer H. P. & Petruccione F. Theory of Open Quantum Systems (Oxford, New York, 2002).

[b28] Liu R. B., Yao W. & Sham L. J. Control of electron spin decoherence caused by electron¨Cnuclear spin dynamics in a quantum dot, New J. Phys. 9, 226 (2007).

[b29] Vandersypen Lieven M. K. *et al.* Experimental realization of Shor’s quantum factoring algorithm using nuclear magnetic resonance, Nature 414, 883 (2001). 11780055 10.1038/414883a

[b30] Raussendorf R. & Briegel H. J. A One-Way Quantum Computer, Phys. Rev. Lett. 86, 5188 (2001). 11384453 10.1103/PhysRevLett.86.5188

[b31] Carvalho A. R. R., Mintert F. & Buchleitner A. Decoherence and Multipartite Entanglement, Phys. Rev. Lett. 93, 230501 (2004). 15601133 10.1103/PhysRevLett.93.230501

[b32] Cywiński Ł., Lutchyn R. M., Nave C. P. & Das Sarma S. How to enhance dephasing time in superconducting qubits, Phys. Rev. B 77, 174509 (2008).

[b33] Benatti F. & Floreanini R., (Eds.), Irreversible Quantum Dynamics, (Springer, Berlin, 2003).

[b34] Gardiner C. W. & Zoller P. Quantum noise: a handbook of Markovian and non-Markovian quantum stochastic methods with applications to quantum optics (Springer, Berlin Heidelberg New York, 2004).

